# Variation in Type A Trichothecene Production and Trichothecene Biosynthetic Genes in *Fusarium goolgardi* from Natural Ecosystems of Australia

**DOI:** 10.3390/toxins7114577

**Published:** 2015-11-05

**Authors:** Liliana O. Rocha, Matthew H. Laurence, Robert H. Proctor, Susan P. McCormick, Brett A. Summerell, Edward C. Y. Liew

**Affiliations:** 1 The Royal Botanic Gardens and Domain Trust, Mrs Macquaries Rd, Sydney, NSW 2000, Australia; E-Mails: lilianarocha@usp.br (L.O.R.); matthew.laurence@rbgsyd.nsw.gov.au (M.H.L.); brett.summerell@rbgsyd.nsw.gov.au (B.A.S.); 2 Mycotoxin Prevention and Applied Microbiology, National Center for Agricultural Utilization Research, US Department of Agriculture, Agricultural Research Service, 1815 North University Street, Peoria, IL 61604, USA; E-Mails: robert.proctor@ars.usda.gov (R.H.P.); susan.mccormick@ars.usda.gov (S.P.M.)

**Keywords:** DNA sequence, phylogenetics, evolution, mycotoxins metabolite profile

## Abstract

*Fusarium goolgardi,* isolated from the grass tree *Xanthorrhoea glauca* in natural ecosystems of Australia, is closely related to fusaria that produce a subgroup of trichothecene (type A) mycotoxins that lack a carbonyl group at carbon atom 8 (C-8). Mass spectrometric analysis revealed that *F. goolgardi* isolates produce type A trichothecenes, but exhibited one of two chemotypes. Some isolates (50%) produced multiple type A trichothecenes, including 4,15-diacetoxyscirpenol (DAS), neosolaniol (NEO), 8-acetylneosolaniol (Ac-NEO) and T-2 toxin (DAS-NEO-T2 chemotype). Other isolates (50%) produced only DAS (DAS chemotype). In the phylogenies inferred from DNA sequences of genes encoding the RNA polymerase II largest (*RPB1*) and second largest (*RPB2*) subunits as well as the trichothecene biosynthetic genes (*TRI*), *F. goolgardi* isolates were resolved as a monophyletic clade, distinct from other type A trichothecene-producing species. However, the relationships of *F. goolgardi* to the other species varied depending on whether phylogenies were inferred from *RPB1* and *RPB2*, the 12-gene *TRI* cluster, the two-gene *TRI1-TRI16* locus, or the single-gene *TRI101* locus. Phylogenies based on different *TRI* loci resolved isolates with different chemotypes into distinct clades, even though only the *TRI1-TRI16* locus is responsible for structural variation at C-8. Sequence analysis indicated that *TRI1* and *TRI16* are functional in *F. goolgardi* isolates with the DAS-NEO-T2 chemotype, but non-functional in isolates with DAS chemotype due to the presence of premature stop codons caused by a point mutation.

## 1. Introduction

*Fusarium* is an economically significant fungal genus with many species that cause crop disease and mycotoxin contamination. Agriculturally important *Fusarium* species have also been isolated from non-cultivated ecosystems, often associated with asymptomatic plants [1,2,3]. *Fusarium goolgardi* is a recently described species isolated from *Xanthorrhoea glauca* (grass tree) in natural ecosystems of New South Wales (NSW), Australia. Isolates were recovered from both asymptomatic (Khancoban, Tumut and Yass regions) and symptomatic plants (Bungonia State Conservation Area) [4], suggesting the possible involvement of *F. goolgardi* in the observed disease symptoms.

A closely related species, *F. palustre*, has been implicated in sudden dieback of smooth cordgrass (*Spartina alterniflora*) in natural ecosystems in North America [5]. *Fusarium palustre* and *F. goolgardi* are both members of the *F. sambucinum* species complex (FSAMSC), a lineage of *Fusarium* that produces trichothecene mycotoxins. Trichothecenes are known to cause contamination of cereal crops and contribute to plant pathogenesis [6,7,8]. Whether *F. goolgardi* is a trichothecene producer has yet to be investigated.

Trichothecenes are products of sesquiterpenoid metabolism, produced by some species of *Fusarium* and other genera in the order Hypocreales [9]. All trichothecenes have the core 12, 13-epoxytrichothec-9-ene (EPT) structure. However, different trichothecene analogues have different patterns of substitution around this core structure. *Fusarium* trichothecenes are often categorised as either type A or type B. Type A trichothecenes have a hydroxyl group (e.g., neosolaniol, NEO), an ester function (e.g., T-2 toxin) at carbon atom 8 (C-8) of the EPT molecule, or no functional group (e.g., 4, 15-diacetoxyscirpenol, DAS) [10,11]. By contrast, all type B trichothecenes have a carbonyl group at C-8. Type A trichothecenes are highly toxic to animals, causing immune disorders, growth retardation, weight loss, pathological changes in liver cells, and death [12]. Furthermore, these toxins can inhibit mitosis and synthesis of nucleic acids and proteins, as well as induce apoptosis [13,14]. In plants, DAS and T-2 toxin can cause chlorosis and inhibit coleoptile and root elongation [15].

In addition to *F. palustre*, *F. goolgardi* is closely related to the type A trichothecene-producing species *F. armeniacum*, *F. langsethiae*, *F. sibiricum* and *F. sporotrichioides* [4]. Molecular genetics and biochemical analyses have revealed that in *F. sporotrichioides*, type A trichothecene biosynthetic genes (*TRI*) occur at three loci [10,11]. The first locus is the 12-gene *TRI* cluster that includes the terpene synthase gene (*TRI5*), P450 monooxygenase genes (*TRI4*, *TRI11*, and *TRI13*), acyl transferase genes (*TRI3* and *TRI7*), and an esterase gene (*TRI8*). Collectively, *TRI* cluster genes are responsible for synthesis of the EPT molecule and one or two structural modifications at C-3, C-4 and C-15 [11,16]. The second locus consists of the acetyl transferase gene *TRI101*, which is responsible for acetylation of the hydroxyl group at C-3 [9,11]. The third locus consists of the P450 monooxygenase gene *TRI1* and the acyl transferase gene *TRI16*, which are responsible for hydroxylation and acylation of C-8 respectively. Together, *TRI1* and *TRI16* are responsible for structural variation at C-8 of type A trichothecenes [11].

Studies of both type A and B trichothecene-producing species indicate that the evolutionary history of the *TRI* loci is complex and does not always reflect the evolution of species in which the loci occur [17,18]. Analysis of the *F. graminearum* species complex (FGSC) indicates that the lack of correlation between species phylogenies and phylogenies based on some *TRI* cluster genes is a result of balancing selection of ancestral *TRI* cluster alleles that confer different type B trichothecene production phenotypes (chemotypes) [17,18,19,20]. Another study revealed a lack of correlation between species phylogenies and *TRI1*-*TRI16*-based phylogenies [9]. Furthermore, the organization of *TRI* genes in a second lineage of trichothecene-producing fusaria, the *F. incarnatum*-*equiseti* species complex (FIESC), suggests a complex evolutionary history of these genes that includes loss, non-functionalization, and translocation within and between *TRI* loci [9].

The objective of this study was to determine whether *F. goolgardi* could produce trichothecenes and to assess the phylogenetic relationships of its *TRI* gene sequences to those in closely related type A trichothecene-producing species. The results revealed two trichothecene chemotypes, provided evidence for the genetic basis of the chemotypes, and suggested that chemotype differences could be representative of different populations within *F. goolgardi*.

## 2. Results

### 2.1. Mycotoxin Analysis

**Figure 1:**
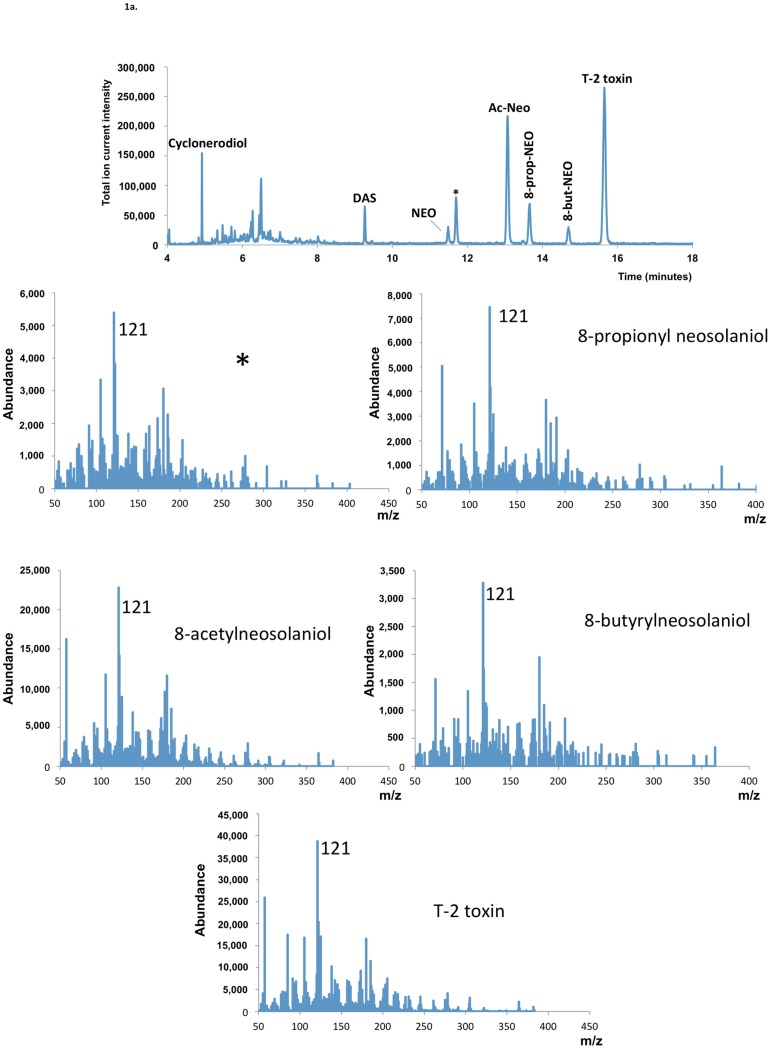

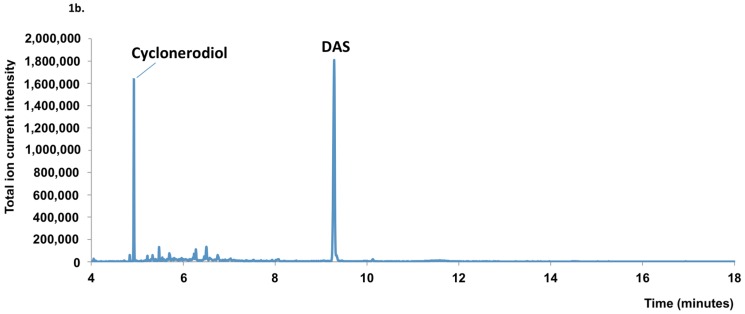
Representative Gas chromatography-mass spectrometry (GC/MS) data generated of *F. goolgardi* culture extracts. (**a**) GC/MS of the *F. goolgardi* RBG5420, representing 4,15-diacetoxyscirpenol-neosolaniol-T-2 toxin (DAS-NEO-T2) chemotype; (**b**) GC/MS of the *F. goolgardi* RBG5421, representing DAS chemotype. * indicates putative 8-acylneosolaniol with MS spectrum similar to those of 8-acetylneosolaniol, 8-propionylneosolaniol, 8-butyrylneosolaniol and T-2 toxin (= 8-isovalerylneosolaniol), *i.e.*, *m*/*z* 121 base peak, and *m*/*z* 364 and 382 ions).

GC-MS analysis revealed the presence of trichothecenes in GYEP culture extracts of all *F. goolgardi* strains examined. The analysis indicated that strains RBG5411, RBG5417, RBG5419, and RBG5420 produced the type A trichothecenes DAS, NEO, 8-acetylneosolaniol and T-2 toxin (=8-isovaleryl neosolaniol) (DAS-NEO-T2 chemotype), whereas isolates RBG5421, RBG5422, RBG6914, and RBG6915 produced only DAS (DAS chemotype) (Figure 1). Neither trichothecenes with a carbonyl group at C-8 (*i.e.*, type B trichothecenes such as deoxynivalenol or nivalenol) nor HT-2 toxin were detected in cultures of any of the *F. goolgardi* isolates. The four isolates with the DAS-NEO-T2 chemotype were from the Bungonia, Khancoban or Tumut region, whereas the four isolates with the DAS chemotype were all from the Yass region (Table S1). Isolates with the DAS-NEO-T2 chemotype also produced 8-propionylneosolaniol, 8-butyrylneosolaniol, and an additional metabolite, which was another type A 8-acylneosolaniol derivative. The other *Fusarium* species used in this study, including *F. palustre*, produced DAS, NEO, Ac-NEO, and T-2 toxin (data not shown).

### 2.2. Sequence Analysis

**Figure 2:**
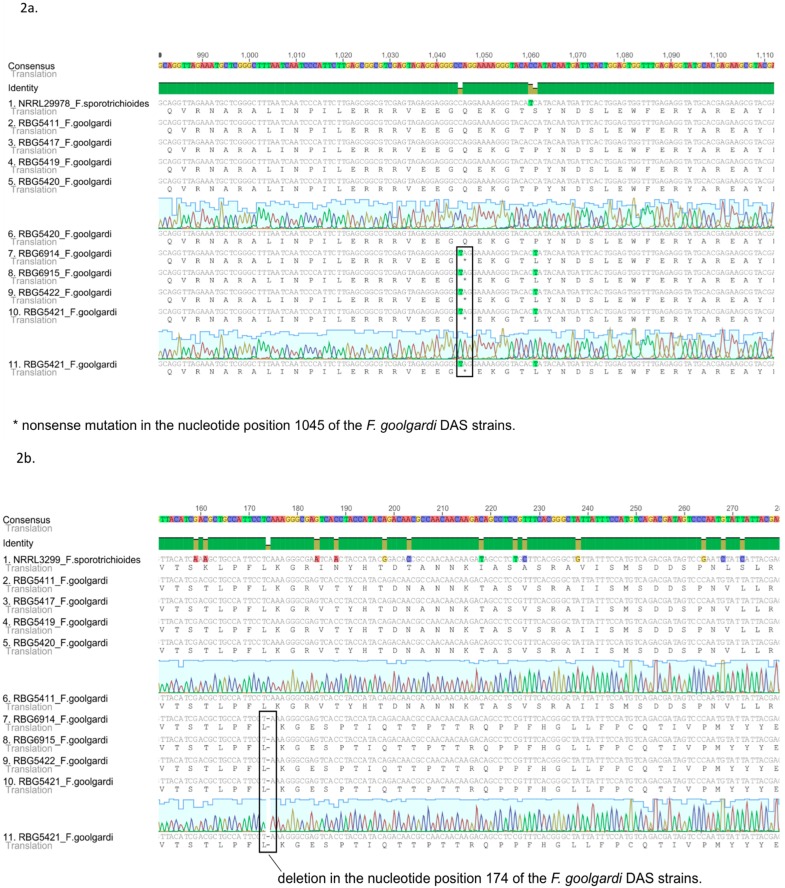

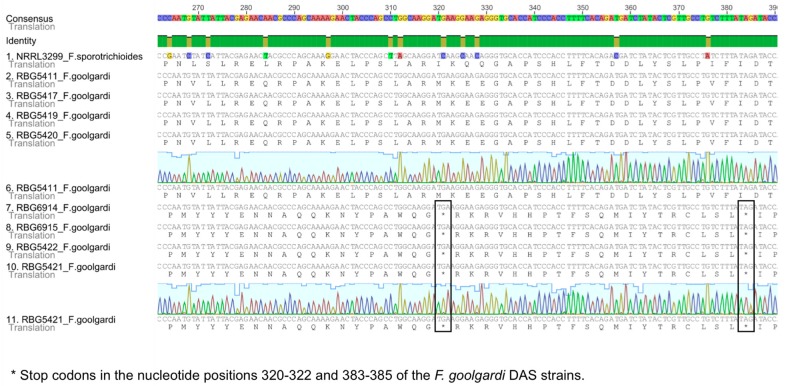
Alignment of the nucleotides and the predicted amino acid sequences of *TRI1* and *TRI16* for *F. goolgardi*. (**a**) Alignment of the nucleotide and the predicted amino acid sequences of *TRI1* from *F. goolgardi* DAS-NEO-T2 and DAS lineages and *F. sporotrichioides* NRRL 29978; (**b**) Alignment of the nucleotide and the predicted amino acid sequences of *TRI16* from *F. goolgardi* DAS-NEO-T2 and DAS lineages and *F. sporotrichioides* NRRL 3299.

Nucleotide sequence data generated in this study for selected *TRI* genes from *F. goolgardi* were aligned against reference sequences from *F. sporotrichioides* strains NRRL 3299 and NRRL 29978. No major differences were observed in the coding region sequences of the two species for the cluster genes *TRI3*, *TRI4*, *TRI5*, *TRI7*, *TRI8*, *TRI11*, and *TRI13* or for *TRI101*. However, sequences of *TRI1*, and *TRI16* in *F. goolgardi* isolates with the DAS chemotype exhibited significant differences from the *F. sporotrichioides* sequences. These isolates exhibited a C-to-T transition that resulted in a premature stop codon (nonsense mutation) in the fourth exon at position 1045 of the *TRI1* coding region (Figure 2). The *TRI16* coding region of DAS-chemotype isolates exhibited a single-nucleotide deletion at position 174, which caused a frame shift mutation and introduced premature stop codons at positions 320–322 and 383–385 of this gene (Figure 2). *TRI1* and *TRI16* orthologs from the other *Fusarium* species examined, including the *F. goolgardi* DAS-NEO-T2 strains, did not exhibit these or any other nonsense or frame shift mutations.

Divergence was observed between isolates of *F. goolgardi* with the DAS or DAS-NEO-T2 chemotype in the sequences of the *TRI* cluster genes, *TRI1*, *TRI16*, and *TRI101*. The number of single-nucleotide polymorphisms (SNPs) observed in the two groups of isolates was 23 for the combined sequences of the *TRI* cluster genes examined, four for *TRI101* and two for *TRI1*. *TRI16* had a single deletion, with no evidence of SNPs.

### 2.3. Phylogenetic Analyses

#### 2.3.1. RNA Polymerase II Largest (*RPB1*) and Second Largest (*RPB2*) Subunits

**Figure 3:**
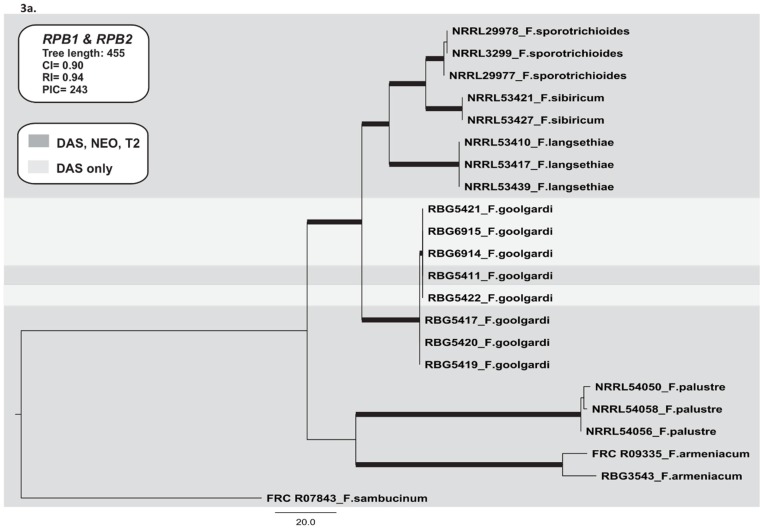

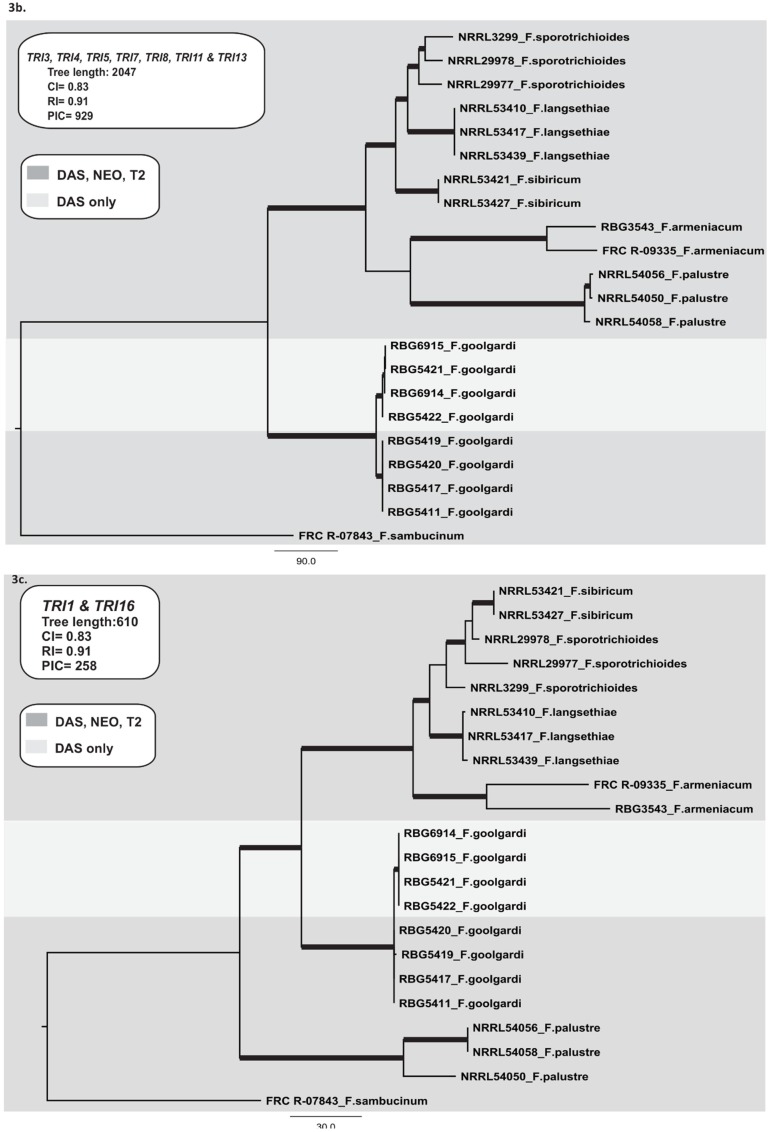

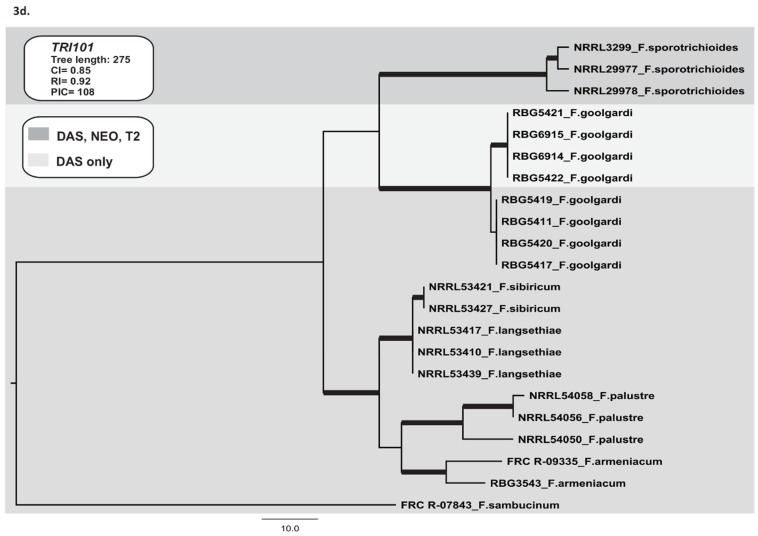
Maximum parsimony trees inferred in this study. (**a**) One of two most-parsimonious trees for the combined *RPB1* and *RPB2* data sets, including 22 isolates with *F. sambucinum* as the outgroup; (**b**) The most parsimonious tree for the combined *TRI* core gene cluster (*TRI3*, *TRI4*, *TRI5*, *TRI7*, *TRI8*, *TRI11*, and *TRI13*), including 22 isolates with *F. sambucinum* as the outgroup; (**c**) The most parsimonious tree for the combined *TRI1* and *TRI16* data set, including 22 isolates with *F. sambucinum* as the outgroup; (**d**) One of ten most-parsimonious trees for *TRI101*. Bootstrap intervals (10,000 replications) greater than 70% and Bayesian posterior probabilities greater than 0.90 are indicated as branches in bold in the phylogenetic trees. The type A trichothecene chemotypes (DAS and DAS-NEO-T2) are indicated by light grey (DAS lineage) and dark grey (DAS-NEO-T2 lineages).

We used sequences of the RNA polymerase genes *RPB1* and *RPB2* to infer a species phylogeny of Type A trichothecene-producing fusaria. The *RPB1* and *RPB2* data set consisted of 22 taxa and 3260 nucleotides with 243 parsimony informative characters (PICs). The analysis resulted in two most parsimonious trees (CI = 0.90, RI = 0.94) (Figure 3a). No major topological variations were detected between trees derived from Neighbour-Joining, Parsimony and Bayesian phylogenetic inference. The species phylogeny was composed of four main lineages: (i) *F. armeniacum*, (ii) *F. goolgardi*, (iii) *F. langsethiae-F. sibiricum-F. sporotrichioides* and (iv) *F. palustre*. The closest relative of *F. goolgardi* was the *F. langsethiae-F. sibiricum-F. sporotrichioides* lineage. Within the *F. goolgardi* clade, a lineage was resolved that consisted of the four isolates with the DAS chemotype and a single isolate with a DAS-NEO-T2 chemotype (Figure 3a). The monophyly of this lineage was not rejected by the SH test (*p* > 0.05) and each lineage was supported by both BPP and MPBS.

#### 2.3.2. *TRI* Gene Cluster

For analysis of *TRI* cluster genes, sequence data for individual genes were concatenated. The resulting data set consisted of 22 taxa and 6750 nucleotides with 929 PICs. The analysis resulted in one most parsimonious tree (CI = 0.83, RI = 0.91) (Figure 3b). No major topological variation was detected between trees derived from Neighbour-Joining, Parsimony and Bayesian phylogenetic inference. The phylogeny included the four main lineages observed in the species phylogeny. However, in contrast to the species phylogeny, the *TRI* cluster phylogeny included a well-supported clade consisting of *F. armeniacum*, *F. palustre* and the *F. langsethiae-F. sibiricum-F. sporotrichioides* lineage, but excluded *F. goolgardi*. Two lineages were resolved within *F. goolgardi*: one consisting of only DAS-NEO-T2 strains, and the other consisting of only DAS strains. There were no major topological differences among trees generated with individual *TRI* cluster genes (*TRI3*, *TRI4*, *TRI5*, *TRI7*, *TRI8*, *TRI11*, and *TRI13*). For the combined data set, SH test did not reject the monophyly of the lineages (*p* > 0.05) for each phylogeny, and all lineages were supported by either BPP or MPBS.

#### 2.3.3. *TRI1* and *TRI16*

The *TRI1–TRI16* data set consisted of 22 taxa and 1933 nucleotides with 258 PICs. The analysis resulted in one most parsimonious tree (CI = 0.83, RI = 0.91) (Figure 3c). No major topological variation was detected between trees derived from Neighbour-Joining, Parsimony and Bayesian phylogenetic inference. The phylogeny consisted of three major lineages: (i) *F. armeniacum-F. langsethiae-F. sibiricum-F. sporotrichioides*; (ii) *F. goolgardi* and (iii) *F. palustre*. In this phylogeny, *F. goolgardi* was resolved as a sister lineage to the *F. armeniacum-F. langsethiae-F. sibiricum-F. sporotrichioides* lineage. In contrast to the other phylogenies, there was no clear separation of *F. sporotrichioides* and *F. sibiricum*, and *F. armeniacum* was closely related to *F. sporotrichioides*, *F. langsethiae*, and *F. sibiricum* with both BPP and MPBS branch support (Figure 3c). A well-supported clade consisting of the four isolates with the DAS chemotype was resolved within *F. goolgardi* (Figure 3c). There were no major topological differences between the *TRI1* and *TRI16* phylogenies. The monophyly of each lineage was not rejected by the SH test (*p* > 0.05), and each lineage was supported by both BPP and MPBS.

#### 2.3.4. *TRI101*

The *TRI101* data set consisted of 22 taxa and 926 nucleotides with 108 PICs. The analysis resulted in ten most parsimonious trees (CI = 0.85, RI = 0.92) (Figure 3d). No major topological variation was detected between trees derived from Neighbour-Joining, Parsimony and Bayesian phylogenetic inference. The phylogeny consisted of five lineages: (i) *F. armeniacum*; (ii) *F. goolgardi*; (iii) *F. langsethiae–F. sibiricum*; (iv) *F. palustre*, and (v) *F. sporotrichioides* (Figure 3d). *F. langsethiae* and *F. sibiricum* were more closely related to *F. armeniacum* and *F. palustre*, instead of *F. sporotrichioides* with both BPP and MPBS branch support (Figure 3d). A well-supported clade consisting of the four isolates with the DAS chemotype was resolved within *F. goolgardi*. In *TRI101* phylogeny, the monophyly of each lineage was not rejected by the SH test (*p* > 0.05).

## 3. Discussion

A previous phylogenetic analysis resolved *F. goolgardi* into a lineage within the FSAMSC that includes *F. armeniacum*, *F. langsenthiae*, *F. sibiricum*, and *F. sporotrichioides*, which are among the few *Fusarium* species that produce the C-8 acylated, type A trichothecene T-2 toxin [4]. The results of the current study demonstrate that some isolates of *F. goolgardi* can produce T-2 toxin, as well as other type A trichothecenes. None of the isolates examined produced type B trichothecenes. Thus, trichothecene production in *F. goolgardi* is similar to its closest relatives for which data is available. The identification of the two chemotypes (DAS and DAS-NEO-T2) among isolates of *F. goolgardi* is unusual among T-2 toxin-producing species of the FSAMSC. Only a single chemotype has been reported for *F. armeniacum*, *F. langsethiae*, *F. sibiricum* and *F. sporotrichioides* [21,22,23,24]. However, in the more distantly related type species *F. sambucinum*, chemotype variation similar to that of *F. goolgardi* was previously reported [25]. Moreover, the *F. goolgardi* DAS-NEO-T2 strains also produced other compounds with mass spectra that indicate that they are other 8-acyl derivatives of NEO. Further investigation is required for complete chemical and characterization of these derivatives.

The presence of premature stop codons within the coding regions of *TRI1* and *TRI16* from *F. goolgardi* isolates with the DAS chemotype almost certainly renders the two genes nonfunctional and is consistent with the lack of production of trichothecenes with modifications at C-8. In *F. sporotrichioides*, *TRI1* and *TRI16* are responsible for hydroxylation and acylation, respectively, of type A trichothecenes at C-8 [26,27,28]. Thus a nonfunctional *TRI16* in *F. goolgardi* should prevent acylation of the C-8 hydroxyl of trichothecenes, and a non-functional *TRI1* should prevent hydroxylation of the C-8. Although a laboratory-induced point mutation responsible for alteration of a type A trichothecene chemotype in *Fusarium* has been reported previously [26], to our knowledge, this is the first report of a naturally occurring point mutation responsible for such an altered chemotype.

The *TRI* cluster, *TRI-TRI16* and *TRI101* phylogenies resolved isolates with the DAS chemotype into a distinct clade within *F. goolgardi*. A similar lineage was resolved in the *RPB1* and *RPB2* phylogeny, but it included one isolate with the DAS-NEO-T2 chemotype. A previous study showed that phylogenies inferred from *TRI* cluster genes and *TRI101* are correlated with the species phylogeny, but the *TRI1* and *TRI16* are not correlated to the species phylogeny [9]. In the current investigation, the *TRI1*-*TRI16*, and *TRI101* phylogenies differed from the species phylogeny. Proctor *et al.* [9] have also suggested that the *TRI1*-*TRI16* locus was the ancestral character state, with at least four different alleles for *TRI1* in the ancestral trichothecene-producing *Fusarium* species [9]. Furthermore, *TRI16* was probably functional in the ancestral *Fusarium* species [9], as it is more likely for a gene to lose functionality than for a gene with multiple deletions and nonsense mutations to become functional [9]. If this is the case, the *TRI1-TRI16* locus in *F. goolgardi* isolates with the DAS-NEO-T2 chemotype is ancestral to the locus in isolates with the DAS chemotype. The results also suggest that further polymorphisms have occurred in the DAS lineage, possibly causing loss of functionality in *TRI1* and *TRI16.* Interestingly, both genes had deleterious mutations within the DAS lineage of *F. goolgardi*. The likely scenario that caused these mutations within *TRI1-TRI16* locus is difficult to determine; however, genome sequencing of *F. goolgardi* may shed more light on this observation.

The *F. goolgardi* DAS lineage was recovered from the Yass region, whilst the DAS-NEO-T2 isolates were recovered from the Bungonia, Khancoban and Tumut regions. Despite the proximity among these regions (about 100 km distance between each region), two chemotypes were observed. This could be indicative of a population subdivision associated with chemotype differences in *F. goolgardi*; however, further surveys in NSW and other regions of Australia where *X. glauca* occurs are required for a better understanding of *F. goolgardi* chemotype and population diversity. In Bungonia, *F. goolgardi* was associated with decline symptoms of *X. glauca* [4]. Considering that fungal toxins contribute to plant pathogenesis [29] and that trichothecene production contributes to the virulence of some fusaria on wheat and maize [30,31], studies should be conducted to evaluate if *F. goolgardi* and type A trichothecenes are involved in *X. glauca* decline in Bungonia National Park. However, it is important to highlight that several environmental factors may influence mycotoxin biosynthesis [32] and various molecules can be involved in *Fusarium* pathogenicity [33,34]. Consequently, pathogenicity tests in *X. glauca* and the characterization of patterns of directional selection in the *TRI* genes would aid in answering these questions.

The findings from this study provide evidence for the genetic basis of trichothecene chemotype variation in *F. goolgardi*, but further investigations are required to verify whether the two chemotypes represent two distinct populations of the fungus. Furthermore, analysis of the pathogenicity and trichothecene production of *F. goolgardi* in planta is warranted to determine whether the fungus and its ability to produce trichothecenes contribute to decline of *X. glauca* in natural ecosystems.

## 4. Experimental Section

### 4.1. Fusarium Isolates

A total of 22 *Fusarium* strains representing the species *F. armeniacum*, *F. goolgardi*, *F. langsethiae*, *F. palustre*, *F. sambucinum*, *F. sibiricum*, and *F. sporotrichioides* of the FSAMSC were selected for analysis (Table S1). The species were selected based on their ability to produce type A trichothecenes [20] and their phylogenetic affinities to *F. goolgardi* [4]. The isolates were obtained from the *Fusarium* Research Centre (FRC); the culture collection at the Pennsylvania State University (State College, PA, USA); the Northern Regional Research Laboratory (NRRL, Peoria, IL, USA); Agricultural Research Service culture collection (Peoria, IL, USA); and the Royal Botanic Gardens (RBG) and Domain Trust collection (Sydney, Australia).

### 4.2. Mycotoxin Analysis

Trichothecene production was analysed by gas chromatography-mass spectrometry (GC-MS). *Fusarium* strains were initially grown on V-8 juice agar for seven days at 25 °C prior to GC-MS analysis. Strains were then transferred to GYEP liquid medium (5% dextrose, 0.1% yeast extract, 0.1% peptone; 20 mL in 50-mL Erlenmeyer flask) and cultivated at 28 °C in the dark at 200 rpm [35]. After seven days, 5 mL culture aliquots were extracted with 2 mL ethyl acetate, dried under nitrogen stream and re-suspended in 200 μL ethyl acetate. GC-MS analysis was performed on a Hewlett Packard 6890 gas chromatograph fitted with a HP-5MS column (30 m length × 0.25 mm internal diameter × 0.25 μm film thickness) and a 5973 mass detector. The carrier gas was helium with 20:1 split ratio and a 20 mL min^−1^ split flow. The column was held at 120 °C at injection, heated to 260 °C at a rate of 20 °C/min and held for 13.4 min [35]. The presence of T-2 toxin, DAS, NEO and other trichothecenes in culture extracts was determined by comparison of retention times and mass spectra of purified toxin standards.

### 4.3. Locus Selection

The housekeeping genes *RPB1* and *RPB2* were selected based on the ability to resolve inter/intra-species nodes within *Fusarium* using the nucleic acid sequences of these genes [4,20,36,37]. *TRI* genes were selected based on the trichothecene biosynthetic pathway and on the previous study by Proctor *et al.* [9]. Hence, the following genes were selected for this study: cluster genes (*TRI3*, *TRI4*, *TRI5*, *TRI7*, *TRI8*, *TRI11*, *TRI13*); as well as *TRI1-TRI16* and *TRI101*.

### 4.4. DNA Extraction, PCR Amplification and Sequence Analysis

*Fusarium* cultures were grown on potato dextrose agar (PDA) for 5 days at 25 °C. DNA was extracted using the FastDNA kit (Q-Biogene Inc., Irvine, USA), according to the manufacturer’s instructions. Primer sets are shown in supplementary Table S2. PCR conditions for amplifying the partial sequences of *RPB1*, *RPB2* [36,37] and *TRI* genes [9] were followed according to their respective protocols. The purified amplicons were sent to the Ramaciotti Centre for Gene Function Analysis at the University of New South Wales where DNA sequences were determined using an ABI PRISM 3700 DNA Analyser (Applied Biosystems, Foster City, CA, USA). Sequences were aligned for each isolate using the multiple alignment program ClustalX v. 1.83 plug-in [38] in the software Geneious v. 5.3.6 (Biomatters, Auckland, New Zealand) [39]. The alignments were edited using the sequence alignment-editing program Geneious v. 1.83 [39] and polymorphisms were confirmed by re-examining the chromatograms.

Similarities of gene sequences detected in this study were verified against the current nucleotide sequences available in the National Centre for Biotechnology Information (NCBI) [40]. The coding regions of the generated sequences were aligned against the annotated reference of *TRI* gene sequences downloaded from NCBI for verifying the presence of nonsense mutations. The sequences generated in this study were deposited in GenBank (Table S1).

### 4.5. Phylogenetic Analyses

The combined *RPB1* and *RPB2* data sets were used to infer a species phylogeny. The *TRI* gene phylogenies for species in the FSAMSC were generated from the following combined data sets: combined *TRI* cluster genes *TRI3*, *TRI4*, *TRI5*, *TRI7*, *TRI8*, *TRI11* and *TRI13*; combined *TRI1* and *TRI16* and *TRI101*. The data sets were combined based on gene location within the three previously described *TRI* loci in *F. sporotrichioides* [9,11] and if the monophyly at the lineage level was not rejected by the Shimodaira-Hasegawa (SH) test [41].

Unweighted Parsimony analysis was conducted by using heuristic search option with 1000 random addition sequences and tree bisection reconnection branch swapping in PAUP 4.0b10 (Sinauer Associates, Sunderland, MA, USA) [42]. Gaps were treated as missing data. The Consistency Index (CI) and the Retention Index (RI) were calculated to indicate the amount of homoplasy present. Neighbour-Joining analysis was performed in PAUP 4.0b10 using an appropriate nucleotide substitution model determined by JModelTest (University College Dublin, Dublin, Ireland) [43]. Clade stability was assessed via Maximum Parsimony Bootstrap Proportions (MPBS) in PAUP 4.0b10, using 1000 heuristic search replications with random sequence addition. The data sets were rooted with *F. sambucinum* as it is considered a suitable out-group [9,20]. Bayesian Likelihood analysis was used to generate Bayesian Posterior Probabilities (BPP) for consensus nodes using Mr Bayes 3.1 [44], run with a 2,000,000-generation Monte Carlo Markov chain method with a burn-in of 10,000 trees. JModelTest was used to determine the substitution evolution model for each gene data sets. The phylogenetic trees were visualised using FigTree v.1.4 (University of Edinburgh, Edinburgh, United Kingdom) [45]. The SH tests performed with PAUP 4.0b10 [41,42] were used to assess the concordance between gene phylogenies [18,46]. Data sets were combined if the different type A trichothecene producing lineages were well supported with MPBS and BPP and if the monophyly at the lineage level was not rejected by the SH test. Monophyly was rejected if the constrained tree log likelihood score was significantly different from the unconstrained topology with 95% confidence level (*p* < 0.05).

## Supplementary Materials

Supplementary materials can be accessed at: http://www.mdpi.com/2072-6651/7/11/4577/s1.
