# Supplementary material for: Variation in Type A Trichothecene Production and Trichothecene Biosynthetic Genes in Fusarium goolgardi from Natural Ecosystems of Australia
Source: Toxins (Basel). 2015 Nov 5;7(11):4577–94. doi: 10.3390/toxins7114577 (PMC4663521; doi:10.3390/toxins7114577)
Supplement: Supplementary file 2 [file toxins-07-04577-s002.pdf]

Supplementary Materials

Table S2. PCR and sequencing primers used in this study.

Gene	Primer Designation	Primer Sequence (5′-3′)	Reference
RPB1	Fa [a]	CAYAARGARTCYATGATGGGWC	O'Donnell *et al.* [37]
	F5	ATGGGTATYGTCCAGGAYTC	
	F7	CRACACAGAAGAGTTTGAAGG	
	F8	TTCTTCCACGCCATGGCTGGTCG	
	G2R [a]	GTCATYTGDGTDGCDGGYTCDCC	
RPB2	5F2	GGGGWGAYCAGAAGAAGGC	O'Donnell *et al.* [37]
	7cR	CCCATRGCTTGYTTRCCCAT	
	7cF	ATGGGYAARCAAGCYATGGG	
	11aR	GCRTGGATCTTRTCRTCSACC	
TRI1	1285	GCGTCTCAGCTTCATCAAGGCAKCKAMTGAWTCG	Proctor *et al.* [9]
	1292	CTTGACTTSMTTGGCKGCAAAGAARCGACCA	
TRI3	1912	TGTGTMGGYGCWGAGGCVATYGTTGG	Proctor *et al.* [9]
	1914	ACRGCAGCRGTCTGRCACATGGCGTA	
TRI4	1450	ACCTTGAGTTCTACCATGAAGTCATC	Proctor *et al.* [9]
	1452	GTTATCCTGTTTGCWGGTACTGAGAC	
	1453	GTCTCAGTACCWGCAAACAGGATAAC	
	1455	ACCTTGAGTTCTACCATGAAGTCATC	
TRI5	1558	GGCATGGTCGTGTACTCTTGGGTCAAGGT	Proctor *et al.* [9]
	1559	GCCTGMYCAWAGAAYTTGCRGAACTT	
TRI7	3870 [a]	GCACSTATKCTGCGCTGATCATC	Proctor, R.H. (unpublished data)
	3871 [a]	CCARATGTAGCCTACAAGCTTGTG	
	3872	GGACGCCAMYGCCGAGCAATGG	
	3873	CCAAAMASCGGCGGCCATTGTTG	
TRI8	3759	GGATTTGAGARYRCYGAGCCTGGAAC	Proctor, R.H. (unpublished data)
	3762	GTGACCTCRTCRKRGATAGCTTGG	
TRI11	1482 [a]	CACACYCTCCTSATGCTYTGTGGACT	Proctor *et al.* [9]
	1483 [a]	TCCCAMACTGTYCTYGCCAGCATCAT	
	1486	GTTGYCTTGAYAACTCKACTTACCA	
	1487	TGCAWYGCCCACATRTCGTTCTCAAC	
TRI13	3874 [a]	CTTGCCGACTTTCGYGAGACVCAAG	Proctor, R.H. (unpublished data)
	3875 [a]	GTCRACCWTGGMCAARCGTTGAAGTGC	
	3876	GGGCTTTGGCTARATCAATTGTTGC	
	3877	CAAGGHGCYGTRTTGGATGCTGT	
TRI16	1472	CCTCTCTCCCCTTGAYCAATTRAACTCT	Proctor *et al.* [9]
	1477	CAATATACGGATACCGCACAAAGACTGG	
TRI101	2067	GGCATYAGCGARGGAAACACAGGA	Proctor, R.H. (unpublished data)
	2068	AGGCTTCTTGGGCATAAAGTACATC	

[a] Primers used for PCR reactions.
